# Off with the label and on the Avastin bandwagon: Why now and how far?

**DOI:** 10.4103/0301-4738.53048

**Published:** 2009

**Authors:** K. S. Santhan Gopal

**Affiliations:** Kamalanethralaya, 302 B Taxila Mansions, CR Layout, ST Bed, Koramangala, 4^th^ Block, Bangalore 560047, India. E-mail: santhangopal@gmail.com

Avastin is used frequently by ophthalmologists. Word search for avastin in the Indian Journal of Ophthalmology website produced 33 articles for the period between 2007 and January 2009, sign of the increased usage of the drug. The reduction of fluid in the retina and improvement in vision after avastin injection is gratifying to all concerned [Figures 1 and 2]. Many with conflicting interests are claimants to this success. There are legal, financial, industrial and ethical implications and the ophthalmologist must be aware of these implications.

**Figure 1:**
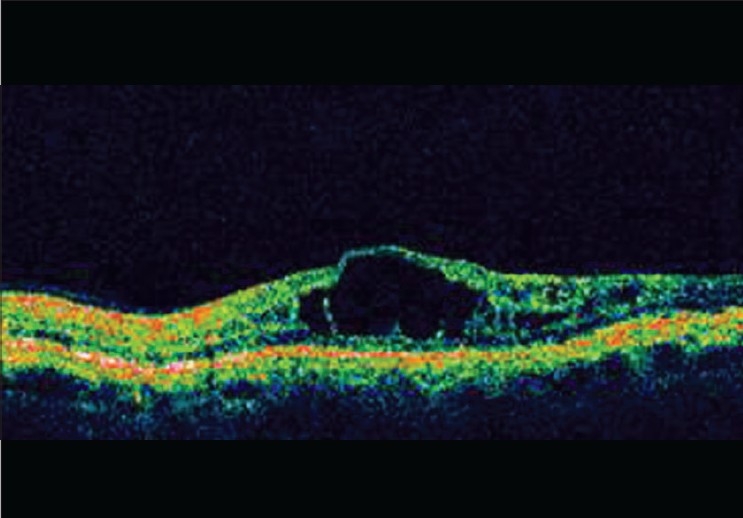
Pre injection Diabetic CME (Courtesy: Dr. Nitin Shetty, Bangalore)

**Figure 2:**
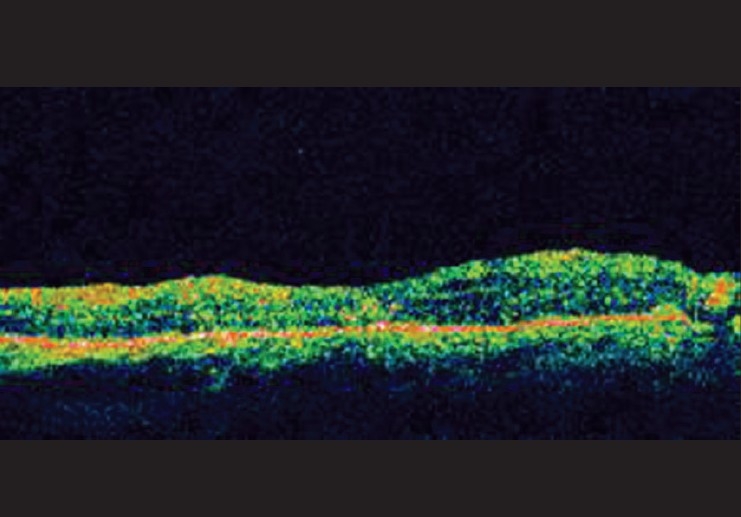
Post Injection

## Legal implications

Bevacizumab (AVASTIN^®^, Genentech, Inc.) was approved by the United States Food and Drug Administration (FDA), for use in tumors for prevention of angiogenesis. The pioneering work of Rosenfeld's[1] with the drug avastin in conditions of angionesis in eyes, led to the use of avastin in many ocular pathologies world over. This work with avastin was done at the time when the related ocular use drugs (like macugen, lucentis) were not available. It's successfully being used intra ocularly for age-related macular degeneration (ARMD) and other conditions like myopic choroidal neovascularization (CNV),[2] sickle cell retinopathy[3] diabetic macular edema and central retinal vein occlusion[4,5] and neovascular glaucoma.[6] Presumably, there will be more indications for its use.

Twenty vitreo-retinal surgeons of India were personally interviewed about the “off label” use of avastin. There was complete agreement about the need for fluorescein angiography and optical coherence tomography (OCT) prior to intravitreal injection, and regular follow-up OCT. Confusion about the legality of “off label” use was significant.

Telephonic conversation with officials at the office of drug controller general, India, in New Delhi, was of no help, as they were not sure of the legal implications of the intravitreal use of avastin.

Before we understand “off-label” use of a drug, we need to understand what a labeled drug is. In the United States a drug is tested in three phases of clinical trials (research studies) before being approved for use on a large scale. The details about the various preclinical phases, clinical phases and ramifications can be found at the site http://www.nlm.nih.gov/services/ctphases.html. [7] In India the drugs are now allowed to enter at the same clinical trial phase as they are in other Western countries without the phase lag. The rules were modified in the year 2005.[8] At the successful completion of a clinical drug trial the Food and Drug Administration (FDA) issues a label to that drug. This is a report of specific information about the drug like the dosage, route of administration, indications contraindications and side effects. The FDA makes this label available to health professionals dispensing and prescribing the drug.

What is an off-label drug?[9] When a drug is used off-label, it is most commonly given for a different disease or medical condition other than described in the FDA-approved label, or it may be given by a different route, or in a different dosage. This is considered off-label use. Off-label is also known as “non-approved” or “unapproved” use of the drug. For example, commonly used subconjunctival injection gentamycin, dexamethasone, intravitreal injection of vancomycin, triamcinolone are all common off -label usages.

Is the use of off-label drug legal? It is legal to use an off- label drug in the United States.[10] In India the rules are either not formulated or vague. The drug control authority in India (Drug controller general, India) would consider the usage of a drug other than as prescribed in the drug label, as not complying with the regulations! Any drug that is used in a way other than the label given by the drug control authority of India, would be considered as a new drug which has to be approved by the authority, before usage in general public. This means that the drug has to go through the clinical trials before being used on the patient. In effect the drug control authority assumes that the intraocular use of avastin by the ophthalmologists is illegal. Though both the doctor and the drug controller general have the patient's interest in mind, their actions are contradictory to each other. Unless we know the letter of the law regarding the off label usage of the drug, we need to be cautious.

**Figure 3:**
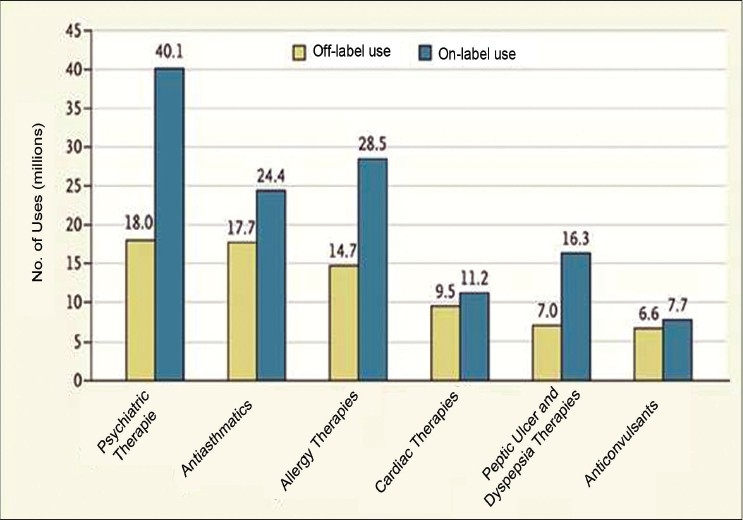
On an average 21% of the drugs used in United States are off label drugs!

On an average 21% of the drugs used in the United States are off label drugs [Figure 3].[10]

While doctors can use drugs for indications other than those approved by the FDA, drug manufacturers themselves cannot peddle their products for such secondary indications.

The doctors can treat their patients with drugs that have not necessarily gone through the rigorous standards of FDA approval. Although approval is indication-specific, the FDA has a limited role once a drug is in the market.

Says Nancy Nielsen, speaker of the American Medical Association's board of delegates “If we only went with FDA-approved uses, science would progress much more slowly. We need to make decisions on medications that are in the best interest of the patient. It's important that physicians get information about off-label drug uses only from peer-reviewed journals. We know very well it is illegal for the sales representatives to discuss them. The whole FDA process may take up to 7 years for a new drug”[11]

With macugen and lucentis, being approved for intraocular use, are we prohibited from using intravitreal avastin?

The answer is in the negative. Drug controllers cannot dictate to the treating physician, what drug should be used, which route etc. Avastin is still legal, though off label and a similar class of drug/s for intraocular use is/are available. Avastin is safe and effective for the time being and presently no change is indicated.

Avastin is the cheapest of the three drugs available for the treatment of ARMD. Remember that the drug has to be repeated and compare the cost here. Table 1, adapted from an article by Azad *et al.*,[12] cost of drug alone for the various modalities of treatment for ARMD.

**Table 1:** Comparison of total estimated cost for different anti-vascular endothelial growth factor drugs

	Cost per dose (Rs.)	Doses expected	Frequency	Total cost (Rs.)
Photodynamic therapy	65000	3	3-monthly	195,000
Macugen	45000	20	4-6 weekly	900,000
Lucentis	65000	20	4-6 weekly	1300,000
Avastin	2000	20	4-6 weekly	40,000

From the point of view of all concerned (patient, physician and the funding agencies) avastin certainly looks the best option. If any other drug is available to treat the retinal conditions, it should be at least comparable in effectiveness and economics to avastin if not better. At least in terms of cost, avastin appears to be the best choice, at present.

## Industry Concerns

The industry feels that it is not very safe to use avastin in lieu of lucentis or macugen. Even assuming that these concerns may be financially inspired, there are certain legitimate worries, that need to be addressed.

At the annual meeting of the American Academy of Ophthalmologists on October 14, 2005, Genentech presented several areas of concern about the drug's potentially adverse effects when used intravitreally for wet ARMD:

1. In view of the fact that avastin contains no preservatives, there could be problems in keeping it sterile during storage and when it is split up by doctors into the small quantities required for retinal treatment there is risk of contamination.
2. No preclinical trial toxicity data exist for use of Avastin in retinal therapy. To go through FDA approval is time-consuming and there is a drug available specifically for intraocular use, why make another?
3. The half-life of avastin is more than lucentis, and so the drug is cleared from the injection site slowly. This may be beneficial in cases of treatment of cancer, but in eyes the avastin being present for a long time, may damage the retina and other ocular tissues.
4. Lucentis binds more strongly to the vascular endothelial growth factor (VEGF) protein than avastin. It is this binding that blocks the protein from developing blood vessel growth in the retina (neovascularization).
5. Avastin contains full-length antibodies, which can cause inflammation. The antibody fragments in Lucentis are one-third the size of avastin antibodies so they are capable of better penetration through the retinal layers.
6. Manufacturing standards differ for cancer and ophthalmic drugs. Particulate matter must be very low in drugs used in the eye, and avastin is not manufactured with that purpose.

Phil Rosenfeld (who is leading the study of Avastin for retinal treatment at the Bascom Palmer Eye Institute in Miami), holds the view that the issue of purity is not a problem in his work.

These questions are to be answered unambiguously:

1. Since avastin has a longer antibody fragment and lesser retinal penetration, is avastin less effective than the two other drugs in treating retinal conditions? A simple no will suffice.
2. Are the systemic thrombotic episodes more often observed with avastin than lucentis/macugen? The answer is no. Since lucentis is a smaller molecule than avastin (see argument above), it could diffuse from the eye into systemic circulation more often and perhaps lead to more thrombotic episodes.
3. In our context, the contamination of the drug during making up of smaller samples is a genuine concern that needs to be properly addressed. Recruiting the help of a compounding pharmacy will minimize the risk of contamination. In this regard the following letter from Roche is worth a look!

Letter from Roche to healthcare professionals dated December 16, 2008. Related to off-label uses in ophthalmology of bevacizumab (avastin) “Reports of eye inflammation, endophthalmitis, and toxic anterior segment syndrome (TASS) following off-label intravitreal use of avastin^®^ (bevacizumab) Hoffmann-La Roche Limited (Roche), in consultation with health Canada, would like to inform you of important new safety information regarding off-label intravitreal use of AVASTIN. It's a recombinant humanized monoclonal antibody that is directed against the VEGF. It is authorized for intravenous administration in the first-line treatment of patients with metastatic carcinoma of the colon or rectum in combination with fluoropyrimidine-based chemotherapy. Use of avastin in the ophthalmology setting has neither been reviewed nor authorized by health Canada. As of November 26, 2008, Roche has been made aware of 25 spontaneously reported Canadian cases of eye inflammation, endophthalmitis, blurred vision, and floaters, some of which have been described as TASS, in patients who were administered aliquots of avastin Lot B3002B028 intravitreally. This is currently the subject of further investigations. All analytical release data has been reviewed by Roche for this manufactured lot and all test parameters were well within limits established for the authorized use of avastin. A review of adverse event reports received in 2008 does not indicate any unusual reporting pattern associated with this lot or any other particular lot of avastin distributed in Canada, when used for the authorized indication. TASS is a sterile postoperative inflammatory reaction caused by a non-infectious substance that enters the anterior segment of the eye and results in toxic damage to intraocular tissues. Roche has neither studied nor sought authorization for the use of avastin in the ophthalmology setting. The current production methods, formulation and dosages for avastin were developed specifically for intravenous use in the oncology setting”.

## Ethical Implications

The patients who cannot afford the higher cost of therapy with lucentis or macugen, may feel that they are being treated with an inferior quality drug. The resultant anxiety needs to be addressed as well.

Unless the clinical superiority of lucentis or macugen over avastin can be clearly demonstrated, the physician should not influence the patients to get these former drugs in preference to the latter.

Insurance companies will dictate to the patients and doctors, as to the drug usage. If the results with lucentis are shown to be clearly better than avastin, we will have a major ethical issue. Retinal receptor atrophy reported as a complication following repeated avastin injections is a matter of serious concern.[13]

So the cautious advice would be to say “off with the label and on the bandwagon” in the interest of the patient. We can continue status quo, until something drastic demands a change.
